# Primary hyperparathyroidism in a patient with primary aldosteronism

**DOI:** 10.1186/s13104-015-1271-0

**Published:** 2015-07-22

**Authors:** Barish Sarıakjali, Esma Jamaspishvili, Mehtap Evran, Murat Sert, Tamer Tetiker

**Affiliations:** Division of Endocrinology, Department of Internal Medicine, Cukurova University Medical Faculty, 01330 Adana, Turkey; Division of Endocrinology, Department of Internal Medicine, Tbilisi State Medical University, 0177 Tbilisi, Georgia

**Keywords:** Primary hyperparathyroidism, Primary aldosteronism, Multiple endocrine neoplasia syndromes

## Abstract

**Background:**

Primary hyperparathyroidism is one of the most common causes of hypercalcemia. Inherited forms of primary hyperparathyroidism like Multiple Endocrine Neoplasia Type 1, Multiple Endocrine Neoplasia Type 2a, Hyperparathyroidism-Jaw Tumor Syndrome or isolated familial tumors are not common for our population.

**Results:**

We present a case of primary hyperparathyroidism in a 38-year-old Turkish man with hyperaldosteronism (Conn’s syndrome).

**Conclusion:**

Genetic studies could not reveal any mutation. We could not identify any inherit form of the diseases. We wanted the first-line relatives examination of the suspected gene mutation, but they refused.

**Electronic supplementary material:**

The online version of this article (doi:10.1186/s13104-015-1271-0) contains supplementary material, which is available to authorized users.

## Background

Primary hyperparathyroidism is usually diagnosed incidentally, few patients have overt signs or symptoms of the classic disease, therefore the disease considered to be asymptomatic. Hypercalcemia and high levels of serum Parathyroid hormone are typical findings. Neck ultrasound and parathyroid scintigraphy are generally used for diagnosis of parathyroid adenoma [1].

Primary hyperaldosteronism, which in 65% is caused by aldosterone-secreting adenoma (Conn syndrome), is one of the reasons of endocrine hypertension [2]. Association of both tumors was reported rarely [3, 4]. We aimed to re-discuss Primary Hyperparathyroidism, Primary Hyperaldosteronism and Multiple Endocrine Neoplasia syndromes in the light of the findings of this rare case.

## Case presentation

A 38-year-old Turkish male was admitted to our outpatient clinic for hypercalcemia. Before entering our clinic, hypercalcemia has been detected in another medical center. History revealed, that he took ace inhibitor in combination with thiazide diuretic for hypertension. Due to the complications, like hypopotassemia this medicine was replaced with calcium channel blockers. In his family history, none of his first degree relatives had hypertension. His father died at 47 years of age due to biliary tract cancer. His three children were diagnosed with West Syndrome. One of them died at the age of 13 months, while the other 9 months of age. His third child is 2 years old and living with the respiratory support equipment. He also has a 13-year-old healthy son. There is no other significant clinical data in his medical history.

On his physical examination: blood pressure—150/90 mmHg, pulse −74 per minute, rhythmic. His general clinical appearance was good. The other systemic and laboratory findings were the following: complete blood count and thyroid function tests were normal: serum Ca level: 10.9 mg/dl (8.8–10.2), inorganic P: 2.4 mg/dl (2.7–4.5), Mg: 2.3(1.6–2.6), albumine: 3.9 g/dl, parathormone (PTH): 200 pg/ml (12–88), Na: 138 mmol/l, K^+^: 2.8 mmol/l, glucose: 85 mg/dl, creatinine: 0.84 mg/dl, alanine transaminase: 24 U/l, white blood cells 8 ML, hemoglobin 14 g/dl, platelet 219 C-reactive protein 0.4 mg/dl (0–0.8), alkaline phosphatase 61 U/l (38–126), 25-OH vitamin D3 22 ng/ml (14–66). Calcium level in 24-h urine: 251 mg/day, inorganic P: 88 mg/day, and K^+^ value in spot urine: 66 mmol/l. Aldosterone level was measured after normalization of his serum potassium level (result: 26.9 ng/dl). Plasma renin activity was <0.15 ng/ml/h. The patient was detected for multiple endocrine neoplasia syndromes. Levels of catecholamine and its metabolites in 24-h urine were normal. In the low dose (1 mg) dexamethasone suppression test, serum cortisol level was suppressed to the normal range. Serum prolactin, thyroid-stimulating hormone, adrenocorticotropic hormone, growth hormone, insulin-like growth factor 1 and calcitonin were normal.

Thyroid ultrasound revealed a 24 × 14 × 12.5 mm hypoechoic nodular lesion at the upper posterior part of the right thyroid lobe, within the thyroid capsule (intrathyroidal parathyroid adenoma?). MIBI (Technetium (99mTc) sestamibi) scintigraphy of parathyroid glands was consistent with the ultrasound finding. Abdominal magnetic resonance imaging revealed 1.6 cm adrenal gland mass consistent with adenoma, cystic lesions in both kidneys with the largest (3 cm in length) in the right kidney and also on this side 11 mm lesion which may be consistent with angiomyolipoma. Pituitary magnetic resonance imaging was normal.

The patient was operated for parathyroid adenoma. At the 3rd day after the operation, serum Ca level was found to be 9.5 mg/dl, inorganic P—3.6 mg/dl and parathyroid hormone—62 pg/ml. Pathological examination of the surgery material revealed parathyroid adenoma. The patient post operatively was followed during the 2 months with spironolactone therapy for his hypertension. This medication was stopped soon after, due to its side effects like erectile dysfunction. Post-surgery, which was conducted for the lesion (1.6 cm) identified on the left adrenal gland revealed adrenal aldosterone producing adenoma. Follow up the adrenal surgery, his blood pressure came to normal ranges and hypokalemia were improved. Blood potassium and calcium levels were within normal ranges. Genetic screening revealed negative mutation of MENIN gene.

## Discussion

In 25 % of the sporadic parathyroid adenoma, deletion is present on the 11th chromosome and is caused by inactivating mutations in the tumor suppressor gene encoding menin. Although Multiple Endocrine Neoplasia Type 1 includes tumors of the parathyroid, anterior pituitary, and pancreatic islets, the parathyroid tumors are far more prevalent than the others; 95% of affected patients eventually develop hyperparathyroidism. In the literature, adrenal adenomas are seen in 25–40%. 95% of the multiple endocrine neoplasia cases occur before 45 years of age [5]. Our patient was 38 years old and association of parathyroid adenoma and Conn syndrome, but his family history were not any findings suggesting multiple endocrine neoplasia. We suspected that this may be sporadically developed syndrome, however the genetic investigation did not reveal the MENIN gene mutation (Additional file 1).

We looked for other similar cases in the clinical literature. You Lim detected Hurthle cell thyroid cancer and meningioma of the brain in addition to parathyroid adenoma and Conn syndrome presented with left adrenal adenoma in a 68-year-old female patient. In this case Ca was 11.6 mg/dl, K^+^ 1.8 mmol/l, parathyroid hormone 82 pg/ml [6].

Carmela also reported a case with both primary hyperparathyroidism and primary aldosteronism (Conn syndrome). She described the case of 68-year-old female with primary aldosteronism with left adrenal adenoma (3 cm in length), and parathyroid adenoma as well. (Ca: 11.2 mg/dl, parathyroid hormone: 240 pg/ml). Similar to our patient, after the surgery, hypertension, serum Ca and K^+^ levels were normalized [7].

In our case, screening for MENIN gene mutation was negative, so we did not consider multiple endocrine neoplasia syndrome.

## Conclusion

The genetic studies have failed to show the expected gene mutation. We could not identify any known inherit form of the disease. Given the rarity of the disease, we decided to examine the patient first-line relatives, but they refused. In conclusion, primary hyperparathyroidism and primary aldosteronism (Conn syndrome) can be seen together in clinical practice and proper management can guarantee a solid compensation.

## Informed consent

Written informed consent was obtained from the patient for publication of this Case Report and any accompanying images. A copy of the written consent is available for review by the Editor-in-Chief of this journal.

## Additional file

Additional file 1: Care checklist (2013) of information to include when writing a case report.
