# Supplementary material for: Primary hyperparathyroidism in a patient with primary aldosteronism
Source: BMC Res Notes. 2015 Jul 22;8:310. doi: 10.1186/s13104-015-1271-0 (PMC4508971; doi:10.1186/s13104-015-1271-0)
Supplement: Additional file 1: — Care checklist (2013) of information to include when writing a case report. [file 13104_2015_1271_MOESM1_ESM.pdf]

CARE Checklist (2013) of information to include when writing a case report

(cc) BY-NC-ND

Topic	Item	Checklist item description	Reported on Page
Title	1	The words "case report" should be in the title along with the area of focus	1
Key Words	2	2 to 5 key words that identify areas covered in this case report.	1
Abstract	3a	Introduction—What is unique about this case? What does it add to the medical literature?	1
	3b	The main symptoms of the patient and the important clinical findings	1
	3c	The main diagnoses, therapeutics interventions, and outcomes	1
	3d	Conclusion—What are the main "take-away" lessons from this case?	1
Introduction	4	One or two paragraphs summarizing why this case is unique with references	2
Patient Information	5a	De-identified demographic information and other patient specific information	2
	5b	Main concerns and symptoms of the patient	2
	5c	Medical, family, and psychosocial history including relevant genetic information (also see timeline).	2
	5d	Relevant past interventions and their outcomes	2
Clinical Findings	6	Describe the relevant physical examination (PE) and other significant clinical findings.	2
Timeline	7	Important information from the patient's history organized as a timeline	2
Diagnostic Assessment	8a	Diagnostic methods (such as PE, laboratory testing, imaging, surveys).	2
	8b	Diagnostic challenges (such as access, financial, or cultural)	2
	8c	Diagnostic reasoning including other diagnoses considered	2
	8d	Prognostic characteristics (such as staging in oncology) where applicable	2
Therapeutic Intervention	9a	Types of intervention (such as pharmacologic, surgical, preventive, self-care)	2
	9b	Administration of intervention (such as dosage, strength, duration)	2
	9c	Changes in intervention (with rationale)	2
Follow-up and Outcomes	10a	Clinician and patient-assessed outcomes (when appropriate)	2
	10b	Important follow-up diagnostic and other test results	2
	10c	Intervention adherence and tolerability (How was this assessed?)	2
	10d	Adverse and unanticipated events	2
Discussion	11a	Discussion of the strengths and limitations in your approach to this case	3
	11b	Discussion of the relevant medical literature.	3
	11c	The rationale for conclusions (including assessment of possible causes)	3
	11d	The primary "take-away" lessons of this case report	3
Patient Perspective	12	When appropriate the patient should share their perspective on the treatments they received	3
Informed Consent	13	Did the patient give informed consent? Please provide if requested	Yes ☒ No ☐
